# The concordance in lesion detection and characteristics between the Anatomical Intelligence and conventional breast ultrasound Scan method

**DOI:** 10.1186/s12880-021-00628-x

**Published:** 2021-06-21

**Authors:** Juan Li, Hao Wang, Lu Wang, Ting Wei, Minggang Wu, Tingting Li, Jifen Liao, Bo Tan, Man Lu

**Affiliations:** 1 Ultrasound Medical Center, Sichuan Cancer Hospital Institute, Sichuan Cancer Center, School of Medicine, University of Electronic Science and Technology of China, No. 55, Section 4, South Renmin Road, Chengdu, China; 2 Breast Surgeons Center, Sichuan Cancer Hospital Institute, Sichuan Cancer Center, School of Medicine, University of Electronic Science and Technology of China, No. 55, Section 4, South Renmin Road, Chengdu, China

**Keywords:** Breast, Ultrasound, Breast imaging reporting and data system, Anatomical intelligence breast

## Abstract

**Background:**

The aim of this study was to investigate the concordance in lesion detection, between conventional Handhold Ultrasound (HHUS) and The Anatomical Intelligence for Breast ultrasound scan method.

**Result:**

The AI-breast showed the absolute agreement between the resident and an experienced breast radiologist. The ICC for the scan time, number, clockface location, distance to the nipple, largest diameter and mean diameter of the lesion obtained by a resident and an experienced breast radiologist were 0.7642, 0.7692, 0.8651, 0.8436, 0.7502, 0.8885, respectively. The ICC of the both practitioners of AI-breast were 0.7971, 0.7843, 0.9283, 0.8748, 0.7248, 0.8163, respectively. The k value of Anatomical Intelligence breast between experienced breast radiologist and resident in these image characteristics of boundary, morphology, aspect ratio, internal echo, and BI-RADS assessment were 0.7424, 0.7217, 0.6741, 0.6419, 0.6241, respectively. The k value of the two readers of AI-breast were 0.6531, 0.6762, 0.6439, 0.6137, 0.5981, respectively.

**Conclusion:**

The anatomical intelligent breast US scanning method has excellent reproducibility in recording the lesion location and the distance from the nipple, which may be utilized in the lesions surveillance in the future.

## Introduction

Breast cancer is one of the most common malignant tumors for women, accounting for 30% of all newly diagnosed cancers in women [1]. Various methods are routinely used for breast cancer screening, such as Mammography, Magnetic Resonance Imaging (MRI), Digital Breast Tomosynthesis (DBT), and Ultrasound (US) [2–5]. Multiple studies have determined that the mammography is the most commonly used method for breast cancer. Nonetheless, the mammography sensitivity decreases in dense breasts due to tissue overlapping. However, mammography is likely to detect multifocal or multicentric cancers [6, 7]. DBT as a complementary technique, may increase the mammography sensitivity, especially in dense breasts. Moreover, DBT is extraordinary susceptible to architectural distortion and speculation. Handheld Ultrasound (HHUS) has always been an attractive supplement to other imaging modalities among breast cancer patients. Distinct advantages of HHUS include wide availability and accessibility, it is a noninvasive, quick, highly sensitive procedure. Furthermore, it is suitable for women with dense breasts [8]. The obvious ultrasound disadvantage is that image quality and interpretation depends dramatically on the scanning experience [9, 10].

Automated breast volume scanner (ABVS) was initially designed to remove the operator dependency [11, 12]. After image acquisition and reconstruction, a physician is in position to make a diagnosis from the stored images in the workstation any time after the ABUS examination [13], which is also able to automatically annotate detected lesions. However, ABVS is more expensive compared to HHUS [14]. Furthermore, it takes longer than HHUS, which requires a trained medical technologist to scan the whole breast for 20 min [15]. Therefore, HHUS is more suitable for clinical practice in the Chinese general population.

Anatomical Intelligence for Breast (AI breast) is a new procedure using Philips EPIQ 7 Evolution 4.0 ultrasound system. It consists of eL18-4 with embedded sensors, tabletop Field Generator, and mattress, information of position and orientation can be translated and displayed through live on-screen for visual mapping during the examination. While performing a regular breast ultrasound examination, it is not necessary to use a body marker or to manually apply annotations for each image capture. Images are stored while performing sweeps, and critical images can be bookmarked for review. Clinical findings can be auto-annotated, and quick orthogonal views of anatomy can be easily retrieved.

The purpose of this study was to explore the consistency between this scanning technique and the conventional ultrasound scan technique considering the detection and diagnosis of breast lesions.

## Materials and methods

### Patient population

**Fig. 1:**
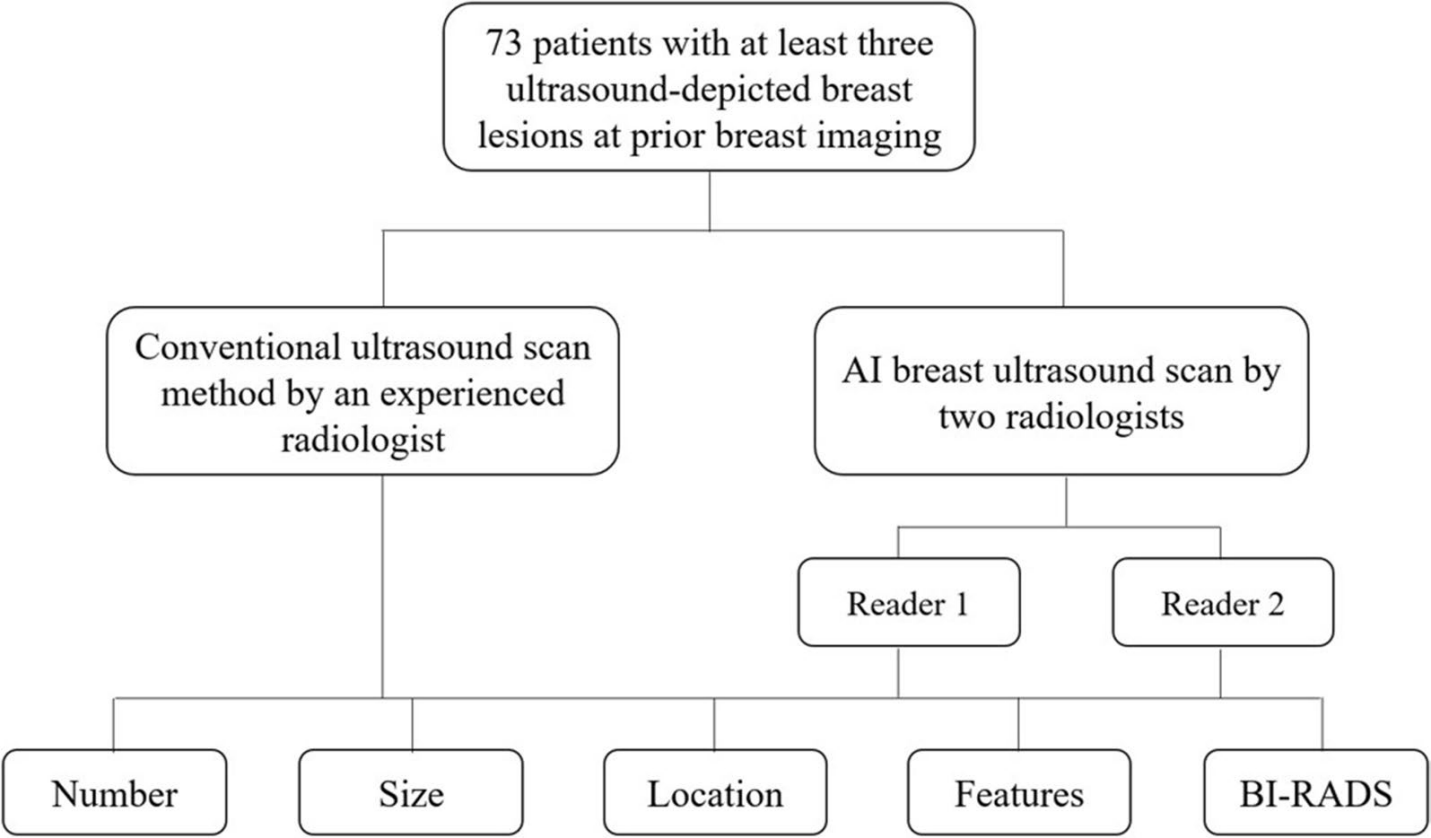
Flowchart of the study

From December 2018 to June 2019, a total of 73 patients with breast lesions were enrolled in this study (Fig. 1), considering that all patients have at least three US-depicted breast lesions at prior breast imaging within a week. All patients underwent HHUS examination by an experienced radiologist (ML). Then all patients underwent AI breast ultrasound examination by a resident physician (JL) and another physician with 5-year experience (BT), respectively. All patients were women between the ages of 20 and 67 (mean 42.6 ± 12.3y). This study was approved by the Institutional Review Board and Ethics Committee of XXX. All patients provided signed informed consent before the examination.

### Equipment

The conventional scan technique and the AI breast scan technique for ultrasound examination was performed using a Philips EPIQ 7 ultrasound system (Philips Medical Systems, Bothell, WA, USA). A 4–18 MHz transducer probe was used for the conventional US, and a 4–18 MHz linear array ultra-broadband transducer with EM tracking was used for the AI breast scan procedure. Tabletop Field Generator and mattress were used for EM tracking.

## Procedure

### Conventional HHUS scan procedure

First, all patients underwent the conventional gray-scale US examination of the breast by an experienced radiologist, who was equipped with over 25 years of experience in breast ultrasound. Patients maintained a supine position and kept arms upward and outward. Investigators were instructed to begin scanning the right breast in a clockwise manner using the lateral and sagittal directions. The characteristics of the lesions were then recorded in line with the demand. In this study, these results assessed by the HHUS were defined as the gold standard (reference standard) as reference with the proposed experimental method. All patients were subsequently examined by wielding AI-breast after obtaining written informed consent.

### AI breast US scan procedure

Each patient underwent AI breast ultrasound examination on the same day by a resident physician and another radiologist with 5 years’ experience, respectively.

**Fig. 2:**
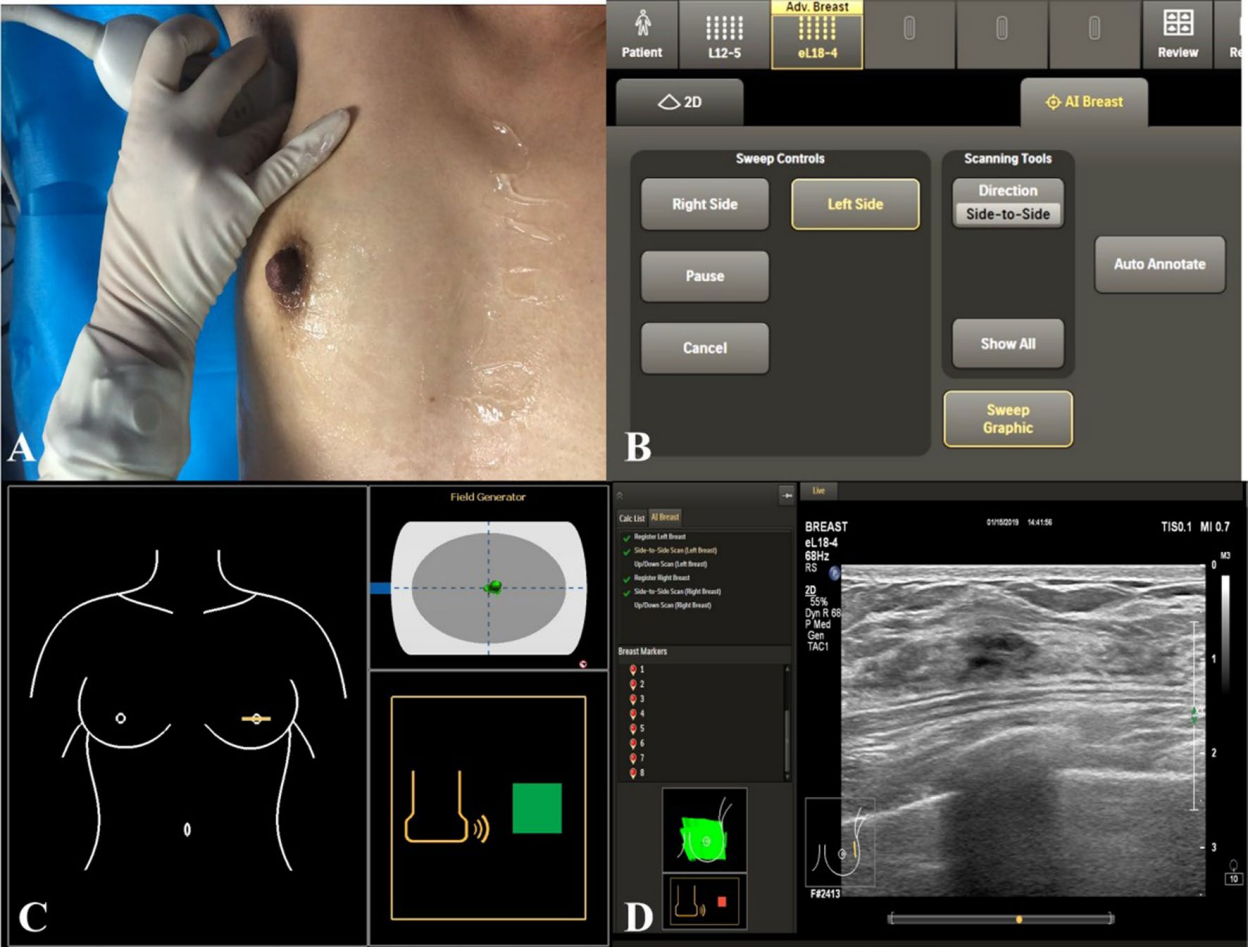
AI breast ultrasound scan method. A -patient maintained a supine position; B- and C- transducer marker verification on the on-screen breast graphic; D- image acquisition

Patients maintained a supine position and kept arms upward and outward. Before scanning, the doctor started from the nipple to ensure that the transducer mark on the breast graphic on the screen was displayed correctly. Before acquiring an image, a quick check was performed to verify that the transducer marker display corresponded to the physical location of the transducer on the breast. When scanning the breast, the transducer on the screen display moves with the scanning hand, keeping the focus on the patient during the scanning process and displaying the image on the screen. When a cine loop is acquired, AI Breast automatically displays and records the transducer position and orientation for each acquisition, and dynamically indicates areas of the breast scanned during the exam, as indicated by the Sweep Graphic. The breast tissue on-screen was assigned a color to demonstrate that it has been scanned in one direction, then assigned a different color once scanned in the opposite plane. (Fig. 2). The characteristics of the lesions were then recorded according to the demand.

During the convention and AI-breast examinations, when lesions are found, the number, the location (clockwise location and distance from the nipple), size (largest diameter and mean diameter), and image characteristics (boundary, morphology, aspect ratio and internal echo) of the lesions are recorded. Ultimately, the diagnosis is made according to the American college of radiology breast imaging reporting and data system, ACR BI-RADS. If the lesions were complex and different, classification diagnosis were considered. The participant recorded the entire scanning time of each patient.

All lesions of BI-RADS 3 were removed by mammotomy biopsy system. Simultaneously, biopsies were performed on all lesions of BI-RADS 4–5.

### Statistical analysis

All data were collected and processed with SPSS 13.0 software (SPSS Inc., Chicago, IL, USA). The enumeration data were statistically analyzed using Chi-square test. Intraclass correlation coefficients (ICCs) have been used to measure agreement on lesion size and location. For the interpretation of κ-values, we made use of the magnitude guidelines published by Landis and Koch, who characterized the values of κ < 0 as indicating no agreement, κ 0–0.20 slight, κ 0.21–0.40 fair, κ 0.41–0.60 moderate, κ 0.61–0.80 substantial, and κ 0.81–1 as almost perfect agreement [13]. The statistical analysis on the extent of agreement between the two raters was based on Cohen's Kappa test. ICCs were calculated for continuous variables. κ statistics were used for measuring agreement on lesion features and final assessments compared with the consensus.

## Results

### Baseline characteristics of the patients

**Fig. 3:**
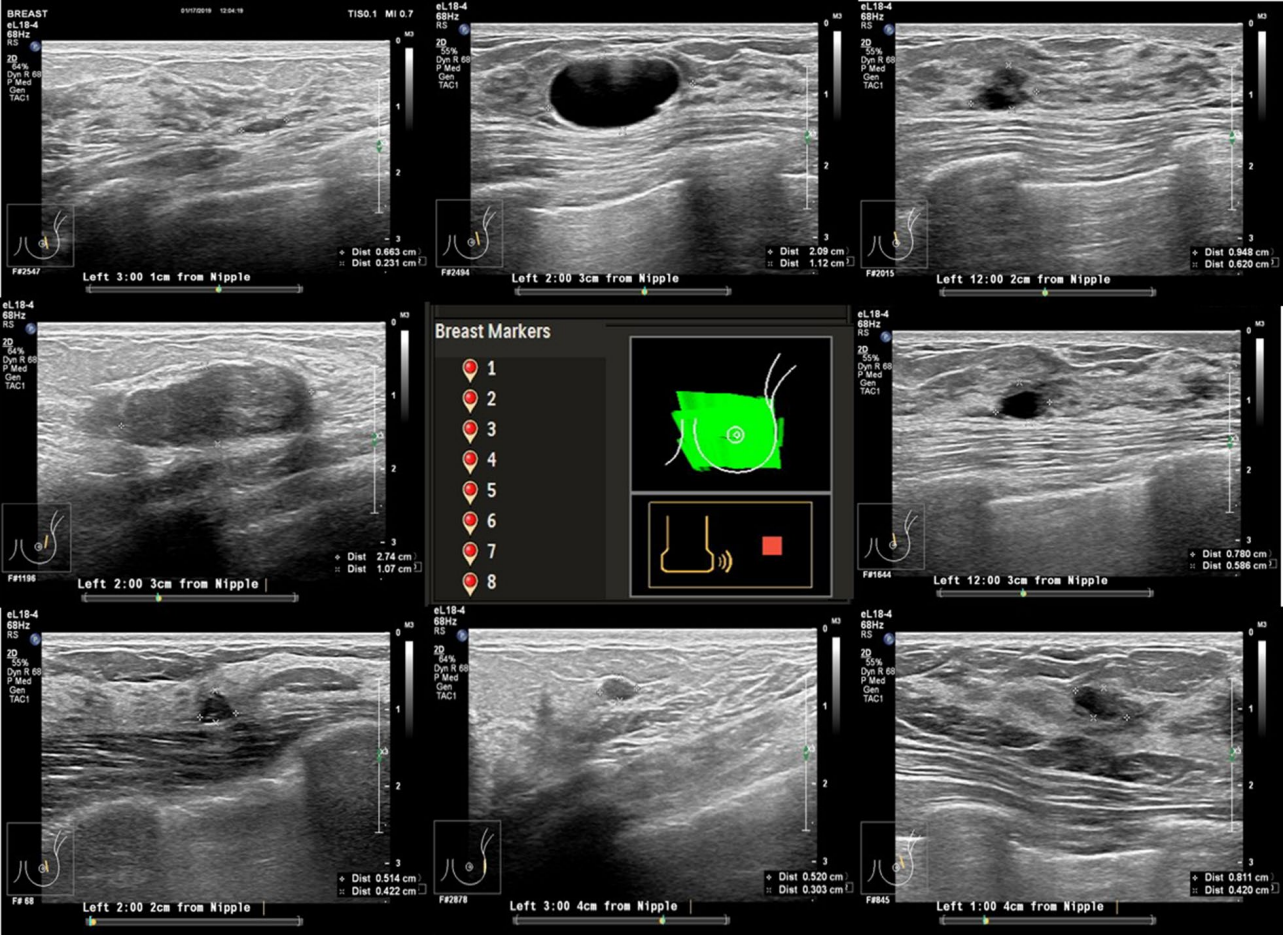
A 45-year-old women presents with eight lesions in the left breast. The number of the breast markers in the left breast and the eight lesions of the left breast

A total of 73 patients were enrolled in this study. 326 lesions were detected by conventional HHUS with a maximum diameter between 4 and 25 mm (mean 8.4 ± 0.78 mm). 299 lesions were detected by a resident physician using AI breast scanning. While 309 lesions were examined by a physician with 5 years experiences using AI breast scanning. A total of 295 lesions (Fig. 3) with a maximum diameter of 2-27 mm and an average diameter (average value of 7.2 ± 0.98 mm). There were 36 lesions classified into BI-RADs 4–5, all of which obtained pathological results by biopsy. All lesions of BI-RADS 3 were excised by mammotomy biopsy system. The remaining lesions classified into BI-RADs 2 were all continuously followed up.

In this study, the ICC between conventional US scan procedure and AI breast US scan procedure in scan time, number, location (clock face location; distance to the nipple), and size (largest diameter; mean diameter) was 0.7642, 0.7692, 0.8651, 0.8436, 0.7502, 0.8885, respectively. The ICC values between reader 1 and reader 2 for scan time, number, location (clock face location; distance to the nipple), and size (largest diameter; mean diameter) in AI breast US scan procedure were 0.7971, 0.7843, 0.9283, 0.8748, 0.8248, 0.8163, respectively. A more detailed breakdown of the concordance between both applications is showed in Table 1.

**Table 1:** Tabulated values for both scanning methods for the scan time, number, location, and size

	C-US	A-US		ICC (C-A)	ICC A1-2
		Reader 1	Reader 2		
Scan time				0.7642	0.7971
Nodule < 5	3.99	4.56	4.21	**0.8995**	**0.9549**
Nodule5-10	6.86	6.25	6.33	0.7417	0.7996
Nodule > 10	10.91	9.26	9.63	0.6664	0.6651
Number	73	73	73	0.7692	0.783
< 5	22	19	20	**0.8015**	0.7982
6–10	36	33	34	0.7652	0.7825
> 10	15	21	19	0.6913	0.8171
Location (Clockface location; Distance to the nipple)					
Clockface location				**0.8651**	**0.9283**
Distance to the nipple				**0.8436**	**0.8748**
Size (Largest diameter; Mean diameter)					
Largest diameter	6.7092	5.9415	6.2764	0.7502	0.7248
1–5 mm				0.4241	0.4531
5–10 mm				0.6538	0.5912
> 10 mm				**0.9614**	**0.9438**
Mean diameter	5.7092	5.9477	6.2437	**0.8885**	**0.8163**
1–5 mm				0.7241	0.7123
5–10 mm				0.6538	0.6759
> 10 mm				**0.9614**	**0.9187**

Data in bold are almost perfect agreement

CUS represents the conventional US scan method; AIUS represents the AI breast US scan method; C-A represents the ICC between the AI breast US scan method and AI breast US scan method; A1-2 represents the ICC between the two reader of AI breast US scan method

In this study, the image characteristics of the two scanning methods between the two readers with AI breast scan procedure including boundary, morphology, aspect ratio, and internal echo, are described in Table 2. The *Kappa* value for image characteristics between conventional US scan method and AI breast US scan method was also shown in Table 2. The ICC between conventional US scan procedure and AI breast US scan procedure in the distinct BI-RADS category in the ABVS examination is also given in Table 2.

**Table 2:** Tabulated values for the image characteristics for both investigated scanning methods

	No. of lesions value			k value		K value sub-feature	
	CUS	AUS-1	AUS-2	C-A	A1-2	C-A	A1-2
Boundary				0.7424	0.6531		
Morphology				0.7217	0.6762		
Aspect ratio				0.6741	0.6439		
Internal echo				0.6419	0.6137		
Cyst	161	132	149			**0.8157**	**0.8756**
Mixed echogenic	66	73	62			0.5132	0.4783
Solid	99	94	98			0.7425	0.7774
BI-RADS assessment				0.6241	0.5981		
2	171	141	148			0.5768	0.5174
3	128	122	130			**0.8243**	**0.8786**
4A, 4B, 4C, 5	27	36	31			0.5132	0.4378

Data in bold are almost perfect agreement

CUS represents the conventional US scan method; AUS represents the AI breast US scan method; C-A represents the k Value between the AI breast US scan method and AI breast US scan method; A1-2 represents k Value between the two readers of AI breast US scan method

## Discussion

Early diagnosis of breast cancer possesses a profound clinical significance in improving the survival rate while reducing the rate of mortality [9, 16]. Ultrasound is one of the commonly used methods for imaging breast lesions [17]. It is convenient, radiation-free and effectively identifies benign and malignant breast lesions [18]. Yet, it is generally necessary to adjust the scanning pressure, focus area and gain in real time to properly analyze the characteristics of the lesion. Meanwhile, the examination of ultrasonography relies on the physician experience and is prone to lead to a missed diagnosis. Furthermore, diagnosing a mass less than 1 cm^3^ is laborious. AI breast is the innovative function of the new generation eL18-4 probe, which can mark the scanning range, automatically identifying the lesion location, improving the ability to locate nodules.

In this study, a substantial agreement for the scan time in two methods (ICC = 0.7642) was found. The average time to complete a bilateral whole-breast US examination between the three readers was 6.663, 7.686 and 6.083, which cannot generate a clinical report immediately. Scan time correlates with the number of lesions. Simultaneously, there was a substantial agreement for the scan time description between the two readers using AI-breast scan method (ICC = 0.7971). The scan time agreement for the two readers using AI breast US is higher than conventional US method, meaning that the AI breast US helps save time, improving departmental efficiency and integrating into the workflow in a busy breast imaging center using virtual mapping.

The substantial agreement for the number of nodules (ICC = 0.7692) between HHUS and AI-breast is valid. Meanwhile, substantial agreement for the number of nodules description between the two readers of AI-breast (ICC = 0.7843) was observed. In this study, we found a substantial moderate consistency was observed between HHUS and AI breast for more than 6 nodules and a substantial consistency between the two readers for AI breast. In the group with more than 10 nodules, AI breast US procedure identified more nodules. This is because, when the number of nodules higher than 6 it is difficult, even for experienced radiologist, to label each nodule using conventional ultrasound. Moreover, AI breast US can mark a lesion in real time in the energetic body to avoid this complication. Radiologists may also miss some breast tissue. While the AI breast US procedure is assigned a color to demonstrate it has been scanned in one direction on-screen, then assigned a different color once scanned in the opposite plane. This helps reduce the operational dependency and subjectivity during exams.

In this study, we also summarized an perfect conclusion for the location of nodules, including clock face location, and distance to the nipple between a resident physician and an experienced radiologist. There was a uniformly perfect agreement for the clock face location, and distance to the nipple between the two readers using the AI breast procedure. This difference between the AI breast two readers is consistent with the coherence between HHUS and AI-breast, where the latter can auto-annotate the lesion with the position and distance from the nipple for the resident physician, allowing for the elimination of labeling errors and enhance reproducibility. This is particularly valuable for the current diagnostic and follow-up examinations when multiple lesions are present.

Breast cancer diagnosis using ultrasound is based on the image features. In this study, a substantial agreement was observed for the lesion features such as boundary, morphology, aspect ratio, and internal echo (k of 0.7424, 0.7217, 0.6741, 0.6419) between both methods. Moreover, substantial agreement between the two readers of AI breast was observed for the same lesion features (k of 0.6531, 0.6762, 0.6439, 0.6137) were similarly moderated. The k value for both methods conveyed consistent results probably due to simple identification in view of a standard acquisition of lesion in AI breast US by resident physicians. AI Breast is capable of not only recording the transducer position and orientation for each acquired lesion, but it can dynamically indicate areas of the breast scanned after the examination.

Based on the comprehensive description of the images above, the consistency evaluation (Kappa) of BI-RADS classification group for lesions by the radiologist and resident physicians connecting AI breast US scan with conventional US scan method was performed. We found a moderate agreement on lesion management, with a k of 0.6241 for BI-RADS final assessments between the two methods and the two readers of AI breast. Previous studies have also reported a moderate agreement in breast US with mammography. This may be because the AI breast US cannot automatically assess the final BI-RADS category. At same time, it was confirmed that the method has excellent repeatability and could reduce the operational dependency and subjectivity during examinations to avoid missed diagnosis.

There were several limitations in our study, such as the long-term follow-up data on the prognoses of these patients, which are still required. Ample and multicenter studies stemming from this study may provide further information for a larger number of patients. Furthermore, the images may be affected by uncertainties and inaccuracies for requiring specific fuzzy image pre-processing steps [19–22].

In summary, AI breast US is less operator-dependent due to the precise documentation of lesion location and distance from the nipple and annotation. In the future, the scanning method can be used to monitor benign lesions since the operator can perform AI breast US examination without identifying the lesion.

## Data Availability

The datasets used and analysed during the current study available from the corresponding author on reasonable request.
